# Exploring Autosomal Dominant Non-Syndromic Monogenic Obesity: From Genes to Therapy

**DOI:** 10.3390/cimb48020162

**Published:** 2026-02-01

**Authors:** Giovanni Luppino, Mara Giordano, Francesca Franchina, Roberto Coco, Eleonora Inì, Carla Fazio, Debora Porri, Cecilia Lugarà, Domenico Corica, Tommaso Aversa, Malgorzata Wasniewska

**Affiliations:** 1 Department of Human Pathology of Adulthood and Childhood, University of Messina, Via Consolare Valeria 1, 98125 Messina, Italy; francesca.franchina8@gmail.com (F.F.); cocoroberto93@gmail.com (R.C.); eleonoraini22@gmail.com (E.I.); fazicarla@gmail.com (C.F.); debora.porri@gmail.com (D.P.); cecilialug@gmail.com (C.L.); domenico.corica@unime.it (D.C.); tommaso.aversa@unime.it (T.A.); 2 Laboratory of Genetics, Struttura Complessa a Direzione Universitaria (SCDU) Biochimica Clinica, Ospedale Maggiore della Carità, 28100 Novara, Italy; mara.giordano@med.uniupo.it; 3 Department of Health Sciences, University of Piemonte Orientale, 28100 Novara, Italy; 4 Pediatric Unit, Azienda Ospedaliera Universitaria—Policlinico Gaetano Martino, Via Consolare Valeria 1, 98125 Messina, Italy

**Keywords:** *MC4R* gene, *SH2B1* gene, *SIM1* gene, *GNAS* gene, *MRAP2* gene, *MC3R* gene, *SRC-1* gene, *KSR2* gene

## Abstract

Genetic factors are key determinants in the pathophysiology of obesity, regulating energy homeostasis. Monogenic non-syndromic obesity accounts for 2–3% of obesity in both children and adults and is most often attributable to mutations in genes encoding components of the leptin–melanocortin pathway. Genetic testing is indicated in children with severe obesity before age 5, hyperphagia, a family history of obesity, and neurodevelopmental delay or organ dysfunction. Mutations associated with monogenic obesity follow autosomal recessive (*LEP*, *LEPR*, *POMC*, and *PCSK1*) or autosomal dominant (*MC4R*, *SH2B1*, *SIM1*, *GNAS*) modes of inheritance. Other gene mutations in heterozygous states (*MRAP2*, *MC3R*, *SRC1*, *KSR2*) are associated with obesity and may exhibit autosomal dominant inheritance; however, the clinical phenotype depends on the degree of genetic penetrance and interactions with other genetic and/or environmental factors. No approved targeted pharmacotherapies are currently available for autosomal dominant monogenic obesity, and the frequent detection of variants of uncertain significance often hinders timely diagnostic confirmation. The review provides a comprehensive appraisal of autosomal dominant forms of monogenic non-syndromic obesity, analyzing genetic and molecular features, clinical presentations, and therapeutic strategies.

## 1. Introduction

**Figure 1:**
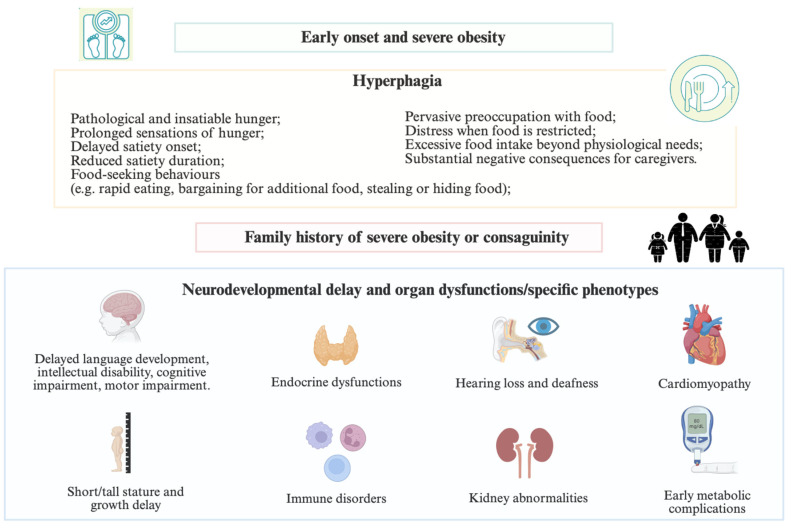
When should genetic obesity be suspected?

Childhood obesity is a chronic, progressive, and relapsing disease, defined as a body mass index (BMI) at or above the 95th percentile [1]. Genetic factors are key determinants in the pathophysiology of polygenic, monogenic, and syndromic obesity by regulating energy homeostasis and the leptin–melanocortin pathway. The most common form of obesity follows a multigenic model of inheritance and involves several multifactorial determinants, in which the cumulative contribution of multiple genes combined with an obesogenic lifestyle result in increased body weight [2,3]. Moreover, pathogenic variants in specific genes represent the main determinants of rare forms of hyperphagia, early-onset, and severe obesity. Monogenic obesity can be further classified into syndromic and non-syndromic forms based on the presence of developmental delay, intellectual disability, or dysmorphic features. Monogenic non-syndromic obesity accounts for approximately 2–3% of obesity cases in both children and adults and is most frequently attributable to alterations in genes encoding components of the central nervous system (CNS) leptin–melanocortin pathway [4,5,6]. Although these conditions are rare, early diagnosis is essential to enable appropriate management strategies and specialist care. Clinical practice guidelines recommend genetic testing in children with severe obesity (≥120% of the BMI 95th percentile) before the age of 5 years, particularly when accompanied by a family history of severe obesity and/or hyperphagia [7,8]. Hyperphagia denotes a pathological and insatiable hunger characterized by persistent food-seeking behaviors [9], leading to significant distress when food is restricted, excessive food intake beyond physiological needs, and substantial negative consequences for caregivers [10,11]. Additional clinical red flags suggesting a genetic cause include neurodevelopmental delay and multisystem disease-specific phenotypes in patients with early-onset and severe obesity. Therefore, specific symptoms and signs, dysmorphic features, organ dysfunction, and early cardiovascular and metabolic complications should be investigated when genetic obesity is suspected (Figure 1) [12,13].

Genetic evaluation represents a crucial step in the diagnostic process of patients with clinical features suggestive of genetic obesity. Single-nucleotide variants responsible for monogenic obesity can be investigated using several genotyping techniques, including Sanger sequencing, short- and long-read next-generation sequencing, and array comparative genome hybridization (aCGH) [14]. Furthermore, advances in genetic knowledge and molecular functional studies are expected to facilitate the interpretation of variants currently classified as variants of uncertain significance (VUS), particularly when correlated with the patient’s phenotype [14,15]. The integration of genomics and advanced technologies into clinical practice holds significant promise for the future management of obesity, including the development of pharmacological options that complement non-pharmacological strategies and target the underlying genetic causes of obesity and hyperphagia. Patients with specific genetic obesity disorders may be treated with available anti-obesity medications, including both targeted and non-targeted agents [13,16]. Targeted pharmacotherapy includes the melanocortin-4 receptor (MC4R) agonist setmelanotide, which has been approved for certain forms of monogenic obesity with autosomal recessive inheritance, as well as for Bardet–Biedl syndrome. Non-targeted anti-obesity medications, such as GLP-1 receptor agonists, naltrexone–bupropion, and incretin-based dual agonists, are approved for obesity and may also be considered in genetic obesity disorders [16].

In monogenic non-syndromic obesity, no approved targeted pharmacotherapies are currently available for variants with autosomal dominant inheritance, and the frequent identification of VUS often hinders timely diagnostic confirmation.

This review aims to provide a comprehensive and critical appraisal of autosomal dominant forms of monogenic non-syndromic obesity. Specifically, variants with predominant autosomal dominant inheritance are extensively analyzed with respect to their genetic architecture and molecular mechanisms, the spectrum of clinical manifestations, diagnostic challenges, and therapeutic perspectives.

## 2. Focus on Leptin–Melanocortin Pathway

**Figure 2:**
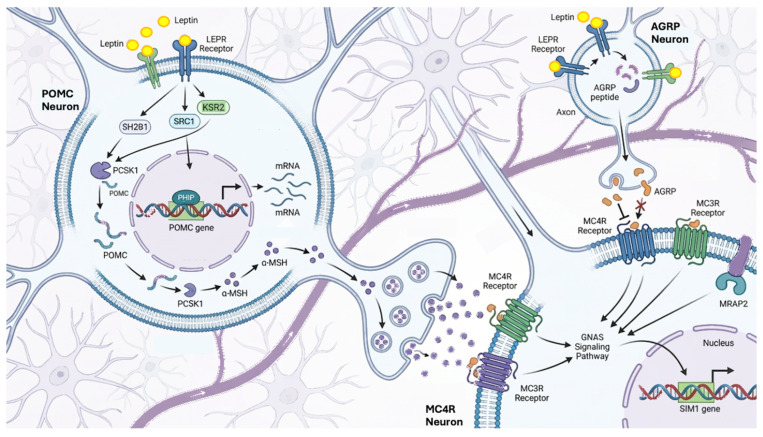
Leptin–melanocortin pathway. AGRP: agouti-related peptide; GNAS: guanine nucleotide-binding protein alpha stimulating; KSR2: kinase suppressor of Ras 2; MC3R: melanocortin-3 receptor; MC4R: melanocortin-4 receptor; MSH: melanocyte stimulating hormone; MRAP2: melanocortin 2 receptor accessory protein 2; SRC1: steroid receptor coactivator-1; PCSK1: prohormone convertase 1; PHIP: pleckstrin homology domain interacting protein; POMC: proopiomelanocortin; SH2B1: src homology 2B adaptor protein B; SIM1: single-minded homolog 1. Figure created in https://scispace.com/, accessed on 18 January 2026.

Monogenic obesity is a neuroendocrine disorder characterized by disruption of the leptin–melanocortin pathway, which plays a pivotal role in the hypothalamic homeostatic regulation of appetite, energy expenditure, and body weight. Within the arcuate nucleus (ARC) of the hypothalamus, two antagonistic neuronal populations are present: anorexigenic proopiomelanocortin (POMC) neurons and orexigenic neurons expressing neuropeptide Y (NPY), agouti-related peptide (AGRP), and γ-aminobutyric acid. These neuronal populations integrate peripheral metabolic cues and project to melanocortin-4 receptor (MC4R)-expressing neurons in the paraventricular nucleus (PVN) [17,18]. Circulating leptin (LEP), derived from adipose tissue, binds to the leptin receptor (LEPR) and activates POMC neurons through LEPR–Src homology 2B adaptor protein (SH2B1)-mediated signaling. POMC is subsequently processed by proprotein convertase 1/3 (PC1/3), encoded by *PCSK1*, into adrenocorticotropic hormone (ACTH) and α- and β-melanocyte-stimulating hormones (αMSH and βMSH), which act on MC4R and melanocortin-3 receptor (MC3R) to promote satiety and increase energy expenditure. In contrast, caloric deprivation activates NPY/AgRP neurons, whose peptides antagonize MC4R, thereby enhancing food intake and reducing energy expenditure [19,20]. These hypothalamic circuits are modulated by additional gastrointestinal endocrine signals, including ghrelin, peptide YY, and cholecystokinin. Proper establishment and function of ARC–PVN connectivity further depend on developmental cues such as class 3 semaphorins (SEMA3) binding neuropilin (NRP) and Plexin-A (PLXNA) receptors, as well as brain-derived neurotrophic factor (BDNF) acting through its receptor NTRK2 (TRKB) [21]. The transcription factor single-minded homolog 1 (SIM1) is essential for PVN development and regulates downstream targets implicated in energy balance, including *MYT1L*, *BDNF*, *TRKB*, and oxytocin [22,23]. Pathogenic variants affecting any component of this pathway impair melanocortin signaling and can lead to early-onset and severe obesity. Genome-wide association studies further support the centrality of this neuroendocrine network, linking numerous variants in genes governing energy metabolism and adipocyte biology to body mass index and obesity risk [13,24]. The leptin–melanocortin pathway is illustrated in Figure 2.

Rare variants associated with obesity follow an autosomal recessive mode of inheritance (*LEP*, *LEPR*, *POMC*, and *PCSK1*). Variants with an autosomal dominant mode of inheritance (*MC4R*, *SH2B1*, *SIM1*, *GNAS*) are causative of monogenic obesity. Other gene mutations in the heterozygous state, including *MRAP2*, *MC3R*, *SRC1*, and *KSR2*, are associated with obesity and a potential autosomal dominant inheritance, although the clinical phenotype depends on the degree of genetic penetrance and interactions with other genetic and/or environmental factors [21]. This review provides a comprehensive overview of genes harboring variants associated with autosomal dominant inheritance.

## 3. Melanocortin 4 Receptor Gene (MC4R)

*Genetic and molecular features*. The *MC4R* gene (OMIM #155541) is an intronless gene with an open reading frame of 999 base pairs, encoding a 332-amino acid protein located on chromosome 18q21.32. It belongs to the melanocortin receptor (MCR) family, which consists of five single-exon genes (*MC1R–MC5R*) encoding G protein-coupled receptors (GPCRs) with diverse physiological roles. The *MC4R* gene is expressed in several tissues, including the hypothalamus, brain, skeletal muscle, adipocytes, and astrocytes. Within the hypothalamus, *MC4R* is prominently expressed in the parvicellular and magnocellular neuronal populations of the paraventricular nucleus (PVN) [25,26]. The *MC4R* gene is a key component of the leptin–melanocortin signaling axis, and its primary functions involve the regulation of food intake, energy homeostasis, and body weight control [27]. Upon leptin stimulation, the α-melanocyte-stimulating hormone (α-MSH) peptide binds to MC4R, ultimately leading to gene expression involved in energy homeostasis. MC4R activation triggers the cyclic adenosine monophosphate (cAMP)–protein kinase A (PKA) pathway, as well as the mitogen-activated protein kinase (MAPK) and extracellular signal-regulated kinase 1/2 (ERK1/2) pathways. These signaling cascades converge in the nucleus to promote cAMP response element-binding protein (CREB)–cAMP response element (CRE)-mediated transcription [28,29,30]. MC4R-induced gene expression influences multiple physiological processes, including the central regulation of food intake and energy homeostasis [30]. In addition, MC4R is involved in the transcriptional regulation of SIM1, a factor known to regulate food intake and to mediate MC4R signaling through a glutamatergic pathway [31].

Mutations in the *MC4R* gene represent the most common genetic cause of monogenic obesity [32]. Autosomal dominant variants account for most cases of monogenic childhood obesity, with heterozygous variants detected in 0.5–5.8% of individuals with early-onset obesity, and prevalence varying according to ethnic background [33]. In non-consanguineous individuals of European origin with obesity, up to approximately 2% may carry pathogenic *MC4R* variants [34]. These mutations exhibit variable penetrance and expressivity, resulting in phenotypes ranging from mild to severe obesity with associated comorbidities [35,36]. In parallel, several common polymorphisms (single-nucleotide polymorphisms, SNPs) in the *MC4R* genomic region have been identified as modifiers of obesity susceptibility in the general population, contributing to multifactorial obesity. A summary of *MC4R* SNPs is reported in Table 1 [37,38,39,40,41,42,43,44,45,46,47,48,49,50,51,52].

Accordingly, two major categories can be distinguished: (1) low-penetrance common polymorphisms, often located in the 3′ region or upstream of the gene, which exert modest but detectable effects at the population level; and (2) rare coding variants (missense, nonsense, or frameshift) that may cause a severe, early-onset obesity phenotype.

*Clinical presentation.* High-penetrance *MC4R* mutations are clinically characterized by hyperphagia, early-onset obesity, increased linear growth, and hyperinsulinemia, with complete loss-of-function mutations associated with a markedly more severe phenotype [32]. The mean age at onset of obesity in children with *MC4R* mutations is approximately 1.2 years (range, 0.25–10 years) in homozygous or compound heterozygous individuals and about 3.8 years (range, 0.16–18 years) in heterozygous individuals [53]. Growth trajectory analyses in monogenic obesity indicate that children with biallelic *MC4R* variants exhibit a pronounced increase in body mass index (BMI) during the first year of life, followed by a relative plateau until approximately 5 years of age. Their linear growth rate increases from around 1 year of age, reaching the ≥97th percentile compared with normal-weight children and roughly the 97th percentile relative to children with common obesity [54,55,56]. In children with monoallelic *MC4R* variants, a steady increase in BMI from birth until 5 years of age is observed, resembling the pattern seen in common obesity. Their linear growth falls within the upper reference range, near the 97th percentile compared with normal-weight children and around the 75th percentile compared with children with obesity [54,57]. Carriers of both classes of variants (monoallelic and biallelic) are taller than individuals with non-genetic obesity after correction for familial predisposition. This acceleration in growth has been correlated with the activity of the growth hormone (GH)–insulin-like growth factor 1 (IGF-1) axis and with hyperinsulinemia, as insulin can activate the IGF-1 receptor [56,57]. *MC4R* variants that limit melanocortin binding to the receptor may act as agonists of the MC3R, stimulating GH release and increasing IGF-1 levels [57,58]. In addition, a significantly larger head circumference has been documented in patients with monoallelic *MC4R* variants compared with patients with common obesity [59]. Data on birth weight, birth length, and growth rate trajectories during puberty remain limited in the literature.

Hyperphagia during meals is a consistent feature in patients with *MC4R* mutations, with greater severity in younger children and in homozygous compared with heterozygous individuals [53]. The C allele of the *MC4R* polymorphism rs17782313 has been associated with abnormal appetite regulation and eating behaviors, resulting in increased energy intake [37,40].

Carriers of *MC4R* mutations do not exhibit a distinctive endocrine profile. No specific alterations in pubertal timing or fertility have been observed in individuals with *MC4R* mutations [53,60]. However, a significant association between polymorphisms in the *MC4R* gene (rs571312 and rs12970134) and precocious puberty in girls has been documented [61]. Pathogenic *MC4R* variants are associated with elevated fasting insulin levels and reduced insulin sensitivity. Nevertheless, a reduction in hyperinsulinemia has been observed with advancing age, consistent with an apparent improvement in hyperphagia during adulthood in these individuals [32,57]. In contrast, *MC4R* deficiency has been associated with significantly lower concentrations of total cholesterol, low-density lipoprotein (LDL) cholesterol, and triglycerides, without affecting high-density lipoprotein (HDL) cholesterol levels. *MC4R* deficiency induces increased expression of the LDL receptor, thereby promoting LDL cholesterol clearance and favoring triglyceride accumulation in adipose tissue due to enhanced clearance of triglyceride-rich lipoproteins and reduced fatty acid oxidation [62]. Systolic blood pressure is reduced in individuals with loss-of-function *MC4R* mutations before the age of 20 years, reaching significantly lower levels than in obese controls. This phenotype results from alterations in sympathetic nervous system tone characteristic of this patient group [62,63,64].

Limited evidence suggests that pathogenic *MC4R* mutations may also be associated with neurobehavioral manifestations and other non-metabolic phenotypes. Mild developmental delays, including language and motor delays, as well as mild intellectual disability, have been reported in some cases [53]. A case of attention-deficit/hyperactivity disorder (ADHD) in an *MC4R* mutation carrier has also been described [65]. Among non-metabolic manifestations, associations between *MC4R* mutations and obstructive sleep apnea syndrome have been reported in a limited number of cases [66].

*Therapeutic Strategies*. Non-pharmacological strategies, including behavioral interventions, lifestyle modifications, and increased physical activity, are less effective in children with *MC4R* deficiency because of persistent hyperphagia, and weight loss is more difficult to maintain in this population [67,68].

Currently, no treatments are specifically approved for individuals with obesity due to *MC4R* mutations. The MC4R agonist setmelanotide has been approved for chronic weight management in patients aged ≥ 6 years with obesity due to pathogenic deficiencies and biallelic variants in *POMC*, *PCSK1*, or *LEPR*, as well as in patients with Bardet–Biedl syndrome (BBS) [69]. Clinical evidence in patients with *MC4R* mutations remains limited and shows heterogeneous results. A clinical trial in heterozygous *MC4R* mutation carriers treated with setmelanotide demonstrated modest weight loss, greater than that observed in individuals with *POMC* deficiency [70].

Regarding glucagon-like peptide-1 (GLP-1) receptor agonists, liraglutide is indicated in adults with obesity (BMI ≥ 30 kg/m^2^) or overweight (BMI ≥ 27 and <30 kg/m^2^) with at least one weight-related comorbidity; more recently, its indication has been extended to children aged 6–12 years with obesity (BMI ≥ 95th percentile) and body weight ≥ 45 kg [71]. In a cohort of patients with monogenic obesity due to pathogenic *MC4R* variants, treatment with liraglutide (3 mg/day for 16 weeks) resulted in an average weight loss of approximately 6% of body weight, comparable to that observed in individuals with non-genetic obesity. Notably, the effects of liraglutide on appetite suppression and weight reduction appear to be preserved even in the presence of *MC4R* dysfunction, suggesting that its mechanism of action may be at least partially independent of MC4R signaling [72]. However, these findings are based on small sample sizes and short-term studies; therefore, their generalizability—particularly to children and adolescents—and the durability of the observed effects require further investigation.

More recently, the dual GLP-1/glucose-dependent insulinotropic polypeptide (GIP) receptor agonist tirzepatide has emerged as an effective therapeutic option in patients with *MC4R* deficiency, demonstrating greater weight loss compared with individuals with a normal *MC4R* genotype [73]. In summary, although the role of setmelanotide in patients with *MC4R* mutations remains to be defined and may be limited to specific responsive variants, GLP-1 receptor agonists and GLP-1/GIP dual agonists currently represent the most promising pharmacological therapeutic options for this population.

Emerging evidence in adolescents suggests that bariatric surgery may constitute the most effective treatment in this age group [74]. The literature does not contraindicate bariatric surgery in patients with *MC4R* mutations; however, in this population, responses appear attenuated and highly variable and may be followed by weight regain in the medium to long term, particularly in individuals with homozygous *MC4R* mutations. These observations suggest that residual receptor functionality and the type of surgical procedure may influence outcomes [75,76,77]. The type of bariatric procedure also appears relevant, with evidence indicating better outcomes following Roux-en-Y gastric bypass (RYGB) compared with sleeve gastrectomy (SG) [75].

In conclusion, bariatric surgery remains a valid therapeutic option for carriers of *MC4R* mutations but requires individualized planning, careful selection of the surgical procedure, long-term follow-up, and continuous interdisciplinary support to optimize outcomes. Medium- to long-term results may also be influenced by the presence of specific *MC4R* polymorphisms.

## 4. Src Homology 2 Domain-Containing Adapter Protein B Gene (SH2B1)

*Genetic and molecular features*. SH2B1 (OMIM #608937), located on chromosome 16p11.2, encodes an intracellular adaptor protein involved in the signaling pathways of various receptor tyrosine kinases and cytokine receptor/Janus kinase (JAK) complexes [78]. SH2B1 is recruited to phosphorylated tyrosine residues on multiple membrane receptor tyrosine kinases, including receptors for leptin, insulin, insulin-like growth factor 1 (IGF-1), and growth hormone (GH). Given the central role of many of these receptors in food intake, energy expenditure, and glucose homeostasis, SH2B1 acts as a critical mediator in the pathogenesis of genetic obesity [79].

Within the leptin–melanocortin pathway, SH2B1 plays an important role in leptin signaling by binding to the long form of the LEPRb and potentiating the activity of Janus kinase 2 (JAK2) [80]. By enhancing downstream signaling pathways, particularly the signal transducer and activator of transcription 3 (STAT3) and phosphatidylinositol 3-kinase (PI3K) pathways, SH2B1 facilitates leptin’s anti-obesity effects. Consequently, impaired leptin signaling is considered the primary mechanism underlying obesity in SH2B1 deficiency. The gene is also highly expressed in the central nervous system (CNS), where it predominantly enhances leptin signaling [81]. Furthermore, SH2B1 functions as an endogenous insulin sensitizer in peripheral tissues, including the liver, skeletal muscle, and adipose tissue. It directly binds to insulin receptors, insulin receptor substrate 1 (IRS-1), and insulin receptor substrate 2 (IRS-2), thereby enhancing insulin sensitivity by promoting catalytic activity and inhibiting tyrosine dephosphorylation of IRS proteins [82].

In humans, both heterozygous loss-of-function (LoF) variants in *SH2B1* and large chromosomal deletions at 16p11.2 encompassing the gene are strongly associated with a phenotype of severe early-onset obesity, hyperphagia, and hyperinsulinemia [83]. Obesity related to *SH2B1* haploinsufficiency most commonly results from the 16p11.2 deletion, a copy number variant (CNV), or from specific LoF point variants within the gene. Deletions in the 16p11.2 region encompassing *SH2B1* represent the most frequent structural genetic cause of severe obesity. The prevalence of this deletion is estimated to be approximately 1 in 3000 individuals in the general population. In cohorts of children with severe early-onset obesity, this rearrangement accounts for approximately 0.5–1% of cases [82,84]. Rare heterozygous LoF point mutations in the *SH2B1* gene, in the absence of the 16p11.2 deletion, have been identified in approximately 0.5–1% of children with severe early-onset obesity [85].

*Clinical presentation.* The clinical phenotype associated with *SH2B1* deficiency is characterized by severe early-onset obesity, coupled with a high prevalence of metabolic and neurodevelopmental comorbidities. Although the 16p11.2 deletion is highly heterogeneous and may involve multiple genes, *SH2B1* is considered the strongest candidate gene responsible for obesity and metabolic phenotype [83].

Patients typically exhibit normal or slightly increased birth weight. The defining clinical feature is the onset of hyperphagia and rapid, accelerated weight gain, which usually becomes evident during the first year of life. Excess weight consists predominantly of fat mass, resulting in severe early-onset obesity. Body mass index (BMI) trajectories are highly predictive, with affected children often showing rapid acceleration in BMI well before the age of 5 years [83]. Data on growth patterns are somewhat conflicting due to the variability of the 16p11.2 deletion syndrome. Although height is frequently reported as normal or within the upper limits of the normal range during childhood, some large cohort studies of patients with *SH2B1* loss-of-function mutations have reported reduced final adult height [86]. This observation is biologically plausible, given that SH2B1 modulates signaling through the growth hormone receptor (GHR) and the insulin-like growth factor 1 (IGF-1) receptor. Due to the severe early-onset obesity phenotype, patients with *SH2B1* haploinsufficiency are at increased risk of developing advanced or precocious puberty, a well-established complication of excess adiposity [87].

The metabolic profile associated with *SH2B1* deficiency is severe and highly penetrant, reflecting the protein’s dual role in leptin and insulin signaling. Leptin resistance represents the central pathogenetic mechanism: patients exhibit elevated leptin levels due to excessive fat mass, but satiety signaling is ineffective as a result of impaired downstream signaling via the LEPRb/JAK2 pathway. Insulin resistance (IR) and hyperinsulinemia arise from the combined effects of obesity and the intrinsic loss of SH2B1 function as an endogenous insulin sensitizer [88]. Given the early onset and severity of IR, features of the metabolic syndrome develop prematurely. The risk of type 2 diabetes mellitus (T2DM) is significantly increased, with onset often occurring earlier than in essential obesity and sometimes manifesting during adolescence. Moreover, polymorphisms in the *SH2B1* gene are strongly associated with T2DM risk in the general population [89]. Non-alcoholic fatty liver disease (NAFLD) is a common and early complication, reflecting severe dysregulation of hepatic lipid metabolism [90]. The coexistence of hyperinsulinemia, dyslipidemia, and hypertension places these individuals at high risk for early cardiovascular disease [91]. In addition, the 16p11.2 microdeletion and microduplication are recognized risk factors for cardiovascular abnormalities, including ventricular septal defects and other forms of congenital heart disease [92]. Mechanistic studies suggest that SH2B1 may be involved in cardiac pathophysiology, possibly through modulation of the JAK2/STAT3 signaling pathway.

Additional endocrine manifestations have been reported in isolated cases of patients with the 16p11.2 deletion, including Hashimoto’s thyroiditis [87], as well as conditions frequently associated with obesity, such as obstructive sleep apnea syndrome (OSAS), vitiligo, enuresis, and strabismus. The most significant non-endocrine complications are related to the 16p11.2 deletion syndrome. *SH2B1* haploinsufficiency may contribute to neurological outcomes through its role in neuronal signaling pathways [93].

Patients frequently present with developmental delay, ranging from global impairment to isolated speech delay. There is a high prevalence of autism spectrum disorder (ASD) and other complex psychiatric and neurological conditions, including schizophrenia [94,95]. Affected individuals often exhibit learning difficulties, behavioral problems (e.g., aggressiveness), and attention-deficit/hyperactivity disorder (ADHD). Studies focusing specifically on *SH2B1* missense mutations have linked these variants to behavioral abnormalities and impaired brain development [96].

*Therapeutic Strategies.* The efficacy of lifestyle interventions alone is limited in most cases of monogenic obesity, including those linked to the leptin–melanocortin pathway. In a case report, a patient with a 16p11.2 deletion achieved successful weight regulation through early parental lifestyle intervention, suggesting that the role of *SH2B1* may not be universally predominant if interventions are initiated early. Furthermore, studies investigating single-nucleotide polymorphisms (SNPs) in the *SH2B1* gene suggest a potential role for nutrigenetics. For example, carriers of the T allele of rs7359397 may derive greater hepatic benefits from energy-restricted Mediterranean dietary patterns [97]. With regard to physical activity, specific genotypes (e.g., *SH2B1* rs7498665) have demonstrated differential effects on body fat composition, indicating that individuals genetically predisposed to weight gain may benefit from higher levels of moderate physical activity [98].

Setmelanotide, a highly affine synthetic agonist of the melanocortin-4 receptor (MC4R), is the first drug approved by the U.S. Food and Drug Administration for the treatment of genetic obesity [99]. Given that *SH2B1* impairment acts upstream of MC4R activation by inhibiting LEPR/JAK2 signaling, direct MC4R stimulation is hypothesized to bypass the underlying defect. Recent clinical trials support this rationale. A one-year trial demonstrated significant clinical benefits in patients with heterozygous *SH2B1* variants or 16p11.2 deletions, with a mean reduction in BMI of up to 9.7% at 12 months and a notable improvement in BMI Z-score among pediatric patients (mean change of −0.55 at 12 months) [100,101]. These findings position setmelanotide as the most effective targeted pharmacological intervention for this condition.

Agents such as semaglutide and liraglutide are also highly promising. Although they do not specifically target the *SH2B1* defect, their mechanisms of action are beneficial for the management of severe obesity, hyperphagia, and associated metabolic complications. Clinical data in patients with *SH2B1* deficiency are still emerging, but the therapeutic rationale is strong. Conversely, because the primary pathogenic mechanism is central leptin resistance due to impaired signal transduction rather than leptin deficiency, treatment with recombinant leptin (metreleptin) is not expected to provide significant benefit in these patients [102].

Finally, bariatric surgery, such as Roux-en-Y gastric bypass (RYGB), remains an option for individuals with severe obesity and life-threatening complications. Although initial weight loss is often substantial, studies in patients with heterozygous variants affecting the leptin–melanocortin pathway, including *SH2B1*, indicate lower overall weight loss and higher rates of weight regain compared with patients with essential obesity [103]. These findings underscore the role of persistent central hyperphagia driven by the genetic defect as a key factor limiting long-term surgical success. Consequently, intensive postoperative nutritional and behavioral support is essential.

## 5. Single-Minded Homolog 1 Gene (SIM1)

*Genetic and molecular features.* Human single-minded homolog 1 (*SIM1*), located on chromosome 6q16.3–q21 [104], encodes a basic helix–loop–helix (bHLH) transcription factor that plays a crucial role in the morphogenesis and functional maturation of specific hypothalamic regions, including the paraventricular nucleus (PVN), the anterior periventricular nucleus (aPV), and the supraoptic nucleus (SON) [105]. Balanced chromosomal translocations and pathogenic missense or frameshift variants in *SIM1* have been associated with hyperphagia and monogenic, non-syndromic, early-onset hypothalamic obesity, typically inherited in an autosomal dominant manner [104,105]. The underlying molecular mechanism involves disrupted hypothalamic development, particularly affecting the PVN and SON, leading to reduced melanocortin-4 receptor (MC4R) expression and decreased numbers of oxytocin (OXT)-, vasopressin (AVP)-, corticotropin-releasing hormone (CRH)-, thyrotropin-releasing hormone (TRH)-, and somatostatin (SS)-producing neurons [106]. Although the downstream transcriptional targets of *SIM1* have not been fully elucidated, the neuropeptide oxytocin has been proposed as a key candidate effector. In mouse models of *SIM1* deficiency, hyperphagia is attenuated by exogenous oxytocin administration and exacerbated by pharmacological blockade of the oxytocin receptor [107]. In a cohort of 2100 pediatric patients with early-onset obesity, 13 heterozygous *SIM1* variants were identified in 28 unrelated individuals; nine of these variants significantly reduced the ability of SIM1 to activate a SIM1-responsive reporter gene [108]. Subsequent functional analyses demonstrated that these variants resulted in reduced SIM1 transcriptional activity, supporting their potential pathogenic contribution to disrupted hypothalamic signaling and early-onset obesity [22].

*Clinical presentations.* Loss-of-function variants in *SIM1* are associated with early-onset, severe, non-syndromic obesity, typically driven by hyperphagia rather than reduced energy expenditure. Mutations in the *SIM1* gene can result in a phenotype partially overlapping with Prader–Willi syndrome (PWS). Bonaglia et al. described patients with overlapping interstitial 6q16 deletions and Prader–Willi-like (PWL) features, including global developmental delay, hypotonia, obesity, hyperphagia, and visual abnormalities. High-resolution array comparative genomic hybridization (array-CGH) precisely mapped all breakpoints, identifying the smallest overlapping deleted region as a 4.1 Mb segment at 6q16.1–q16.2, which likely contains the genes responsible for the PWS-like phenotype [109], including *SIM1* [110]. Functional luciferase assays demonstrated that specific *SIM1* variants (p.T46R, p.H323Y, p.T714A) markedly impaired SIM1 transcriptional activity and were associated with familial obesity, thereby confirming *SIM1* as a significant human obesity gene [111]. Variants in *SIM1* have also been associated with pituitary hormone deficiencies. Hypopituitarism was reported in a 21-month-old boy with early-onset obesity, presenting with central hypothyroidism, central adrenal insufficiency, and partial diabetes insipidus due to a novel SIM1 mutation (c.214C>T, p.Pro72Ser) [112]. The *SIM1* frameshift variant p.Asp98Argfs*29 was identified in a male patient with early-onset obesity, hypothalamic dysfunction, partial diabetes insipidus, transient metabolic complications, and neurobehavioral disorders. In this case, the *SIM1* mutation co-occurred with three additional variants identified through a next-generation sequencing (NGS) obesity gene panel. These findings suggest that pituitary function should be systematically assessed in patients with severe obesity and underlying genetic abnormalities, particularly when *SIM1* mutations are detected [113]. Beyond severe early-onset obesity, *SIM1* mutations that markedly reduce transcriptional activity are associated with a spectrum of neurobehavioral abnormalities. Reported manifestations include cognitive impairment, reduced attention span, emotional lability, and memory deficits [108,114].

*Therapeutic Strategies.* Management of *SIM1*-related obesity currently relies primarily on behavioral modification and nutritional interventions. To date, no gene-targeted therapy specifically addressing *SIM1* deficiency has been approved. Ongoing clinical trials are evaluating the efficacy of melanocortin-4 receptor agonists in patients with genetic forms of obesity, including *SIM1* haploinsufficiency (ClinicalTrials.gov IDs: NCT04963231, NCT05093634). Preliminary data suggest improvements in hyperphagia and the promotion of sustained weight reduction in this patient population, supporting the potential of a precision medicine approach for *SIM1*-associated obesity.

## 6. Guanine Nucleotide-Binding Protein Alpha Stimulating Gene (GNAS)

*Genetic and molecular features.* The *GNAS* gene (OMIM #139320), located on chromosome 20q13.32, is a highly complex imprinted locus that encodes multiple proteins through the use of alternative promoters and extensive alternative splicing. Among these products, the Gsα protein plays a critical role in G protein-coupled receptor (GPCR) signaling by mediating intracellular responses to hormones and other extracellular signals [115]. Notably, the *GNAS* locus exhibits tissue-specific parent-of-origin-dependent expression, whereby either the maternal or paternal allele is epigenetically silenced in certain tissues. Disruption of this finely regulated balance—through either coding-region mutations or epigenetic alterations such as aberrant methylation—can result in distinct disease phenotypes [115,116]. Specifically, pseudohypoparathyroidism type 1A (PHP1A) is caused by maternal loss-of-function mutations affecting exons 1–13 of *GNAS*, leading to parathyroid hormone (PTH) resistance and features of Albright hereditary osteodystrophy (AHO), including short stature and obesity. In contrast, pseudopseudohypoparathyroidism (PPHP) results from similar *GNAS* mutations on the paternal allele and typically presents with AHO features in the absence of hormone resistance. Additional *GNAS*-related disorders include autosomal dominant and sporadic pseudohypoparathyroidism type 1B (AD-PHP1B and spor-PHP1B), which are primarily associated with epigenetic defects [116]. GNAS mutations can also disrupt melanocortin-4 receptor (MC4R) signaling, thereby contributing to hyperphagia, impaired sympathetic nervous system activation, and accelerated growth and weight gain, all of which promote the development of severe obesity. In PHP1A, obesity is specifically linked to maternal Gsα mutations and often manifests in early childhood, leading to significant metabolic and clinical complications. Despite growing insights into the molecular mechanisms of obesity, comprehensive data on the spectrum and frequency of pathogenic variants and genotype–phenotype correlations—particularly involving GNAS in children with severe obesity—remain limited [117]. Importantly, severe obesity may develop in patients carrying *GNAS* missense mutations even before the appearance of other classical features of PHP, especially in cases involving maternal Gsα mutations [117]. In a large cohort of 2548 children with severe obesity, 22 individuals carrying *GNAS* mutations were identified, most of whom exhibited impaired MC4R signaling [118]. Beyond MC4R, Gsα mutations may also affect signaling through other GPCRs involved in metabolic regulation, including β_2_- and β_3_-adrenergic receptors, which play key roles in thermogenesis and lipolysis, as well as the corticotropin-releasing hormone (CRH) receptor. Through these mechanisms, *GNAS* dysfunction may alter sympathetic tone, energy balance, and overall metabolic homeostasis [119]. Overall, *GNAS* mutations occur at a low frequency (typically <1%) in cohorts enriched for monogenic severe obesity, indicating that *GNAS*-mediated obesity represents a rare but clinically relevant genetic etiology [117].

*Clinical presentation.* Patrick Hanna et al. described genotype–phenotype correlations in a cohort of 306 patients with GNAS mutations (PHP1A/PPHP) and 220 patients with epigenetic alterations consistent with PHP1B. Individuals with PPHP, who carry paternal-allele mutations, were smaller at birth and exhibited a significantly reduced mean birth length compared with controls. Short adult stature was observed in 64% of patients with PHP1A and in 59% of those with PPHP. In contrast, patients with PHP1B displayed a distinct growth pattern characterized by accelerated early growth, with height above the population mean at 1 year of age, followed by reduced growth velocity, ultimately resulting in normal adult stature [120]. Additionally, impaired growth was identified in six obese children carrying *GNAS* mutations, attributed to defective growth hormone-releasing hormone (GHRH) receptor signaling and a potential insufficiency of the growth hormone (GH) axis. With respect to body weight, carriers of *GNAS* mutations exhibited a very early onset of obesity, frequently manifesting within the first years of life [118].

In classical *GNAS*-related disorders, including PHP1A and PHP1B, multiple hormone resistance is a well-recognized feature. This typically involves resistance to parathyroid hormone (PTH), resulting in hypocalcemia and phosphate abnormalities; resistance to thyroid-stimulating hormone (TSH), leading to hypothyroidism; and, in some cases, resistance to GHRH with consequent impairment of the GH axis, as well as gonadotropin resistance [121]. Patients harboring frameshift or nonsense variants may also present with subcutaneous ectopic ossifications, a hallmark of the Albright hereditary osteodystrophy (AHO) phenotype [117].

*Therapeutic strategies.* Treatment primarily consists of lifestyle interventions, including a hypocaloric diet and regular physical activity. Although specific therapies remain in early stages of development, the recent literature highlights promising approaches such as MC4R agonists. Given that many *GNAS* mutations impair MC4R signaling, the authors of a study involving 2548 children suggest that patients with *GNAS*-mediated obesity may benefit from MC4R agonist therapy [118]. More recently, a novel compound, Isomeranzin (ISM), has been identified that activates GNAS–AMPK signaling, promotes the browning of white adipose tissue, and reduces obesity in experimental models. These findings indicate that direct modulation of *GNAS* may represent a viable therapeutic strategy in the future [122].

## 7. Melanocortin Receptor Accessory Protein Gene (MRAP1-2)

*Genetic and molecular features.* MRAP1 and MRAP2 encode transmembrane accessory proteins that are crucial for the regulation of melanocortin receptors (MCRs). MRAP1, located at 21q22.11, is involved in the transport and activation of the melanocortin-2 receptor (MC2R), both of which are expressed in the zona fasciculata of the adrenal gland. MRAP1 plays a key role in adrenocorticotropic hormone (ACTH) binding and glucocorticoid production. Consequently, loss-of-function mutations in MRAP1 result in adrenal insufficiency, leading to familial glucocorticoid deficiency (FGD) type 2 [123].

MRAP2 (OMIM #609196), located on chromosome 6q14.3, encodes a 205-amino acid membrane protein expressed in human adrenal and brain tissue. Similarly to *MC4R*, MRAP2 is expressed in regions involved in appetite regulation, including POMC and AgRP neurons. MRAP2 deficiency causes obesity through a mechanism that is not yet fully understood but is likely related to abnormal modulation of the MSH–MC4R pathway. Reduced expression of MRAP2, and consequently an abnormal MC4R/MRAP2 ratio (e.g., 4:1), decreases cAMP production in response to MSH, thereby disrupting melanocortin signaling [124]. In a cohort of 9418 subjects, 23 monoallelic variants of *MRAP2* were identified and functionally tested. Seven of these variants (c.5–5del, c.3–7del, p.Gly31Val, p.Phe62Cys, p.Asn77Ser, p.Lys102X, and p.Pro195Leu) reduced cAMP production in response to MSH and were associated with obesity and abnormal eating behaviors [125]. Furthermore, studies in *MRAP2* knockout (MRAP2^−/−^) mice indicate that severe obesity develops without an increase in food intake compared with wild-type controls, despite equal nutrition, energy expenditure, and respiratory exchange ratio [126].

*Clinical presentations.* Monoallelic variants of *MRAP2* are associated with early-onset obesity and hyperphagia. The clinical phenotypes of patients carrying *MRAP2* mutations differ from those observed in individuals with *MC4R* variants. Obesity in patients with *MRAP2* variants is frequently accompanied by hypertension, hyperglycemia, and altered adrenal function [127]. *MRAP2* enhances ghrelin signaling through its receptor (GHSR-1a), which has been implicated in blood pressure regulation. Further investigation of the interaction between MRAP2 and ghrelin signaling is needed to elucidate the hypertensive phenotype observed in obese patients harboring MRAP2 mutations [128]. Hyperglycemia, glucose intolerance, and IR have also been reported in *MRAP2* knockout mice [129]. Beyond its role in glucose homeostasis and insulin sensitivity, MRAP2 is involved in cardiovascular autonomic regulation, and its dysfunction may contribute to autonomic abnormalities [91]. Additionally, a rare nonsynonymous variant, p.A40S, was identified in a patient with Prader–Willi-like (PWL) syndrome during a screening of the 6q14.1–6q16.3 region in individuals exhibiting PWL phenotypes [110].

*Therapeutic Strategies.* Bariatric surgery was performed in a 24-year-old male patient with severe obesity, hypertension, and hyperglycemia, who carried a pathogenic heterozygous loss-of-function variant in the *MRAP2* gene (p.G52R). The patient achieved a 31% reduction in body weight one year after sleeve gastrectomy [130]. Further data regarding auxological parameters, growth rate, pubertal development, endocrine dysfunction, additional phenotypes, and responses to anti-obesity medications are not yet available in the scientific literature for *MRAP2* gene-related obesity.

## 8. Melanocortin-3 Receptor Gene (MC3R)

*Genetic and molecular features.* MC3R (OMIM #155540) is a class A G-protein–coupled receptor encoded by a single-exon gene located on chromosome 20q13.2–q13.3. MC3R is widely expressed in both the central nervous system (CNS) and peripheral tissues. In the CNS, it functions as an inhibitory autoreceptor on POMC neurons [131]. Functionally, MC3R displays high affinity for γ-melanocyte-stimulating hormone (γ-MSH), suggesting receptor-specific regulatory roles within the melanocortin signaling network. Upon ligand binding, MC3R couples to Gs proteins, activates adenylyl cyclase, and stimulates cAMP production, thereby contributing to the regulation of energy homeostasis, hunger signaling, and sexual maturation [132]. *MC3R* expression has also been detected in human adipose tissue, where receptor levels are modulated by metabolic hormones. Leptin suppresses MC3R expression in adipocytes, whereas ghrelin influences MC3R signaling indirectly via neuropeptide Y receptor-mediated pathways [133]. Additionally, MC3R activity plays a critical role in hepatic autophagy and metabolic regulation, directly affecting liver steatosis and systemic adiposity [134].

In humans, both common polymorphisms and rare pathogenic *MC3R* variants have been reported. The p.Thr6Lys/p.Val81Ile haplotype is the most prevalent variant. In a cohort of 355 children, co-inheritance of these polymorphisms was observed in 8.2% of overweight subjects. These individuals exhibited significantly higher BMI, increased fat mass, and elevated leptin and insulin levels compared with children carrying the wild-type allele or single variants. The combined polymorphisms markedly reduced receptor binding and signaling, whereas single variants had minimal functional impact, indicating that partial MC3R inactivation predisposes to pediatric-onset obesity [135]. Three novel heterozygous mutations (p.Ile183Asn, p.Ala70Thr, and p.Met134Ile) have been associated with increased adiposity and elevated leptin levels, accompanied paradoxically by reduced hunger [136].

*Clinical presentations.* Individuals carrying the common p.Thr6Lys/p.Val81Ile haplotype demonstrate higher body fat percentage, elevated leptin levels, and altered insulin sensitivity [137]. Beyond adiposity, large-scale genomic analyses have linked loss-of-function *MC3R* variants to reduced childhood growth, lower adult lean mass, and delayed pubertal onset, highlighting a broader role for MC3R in human developmental and metabolic regulation [138]. Experimental studies in animal models show that loss of MC3R function results in a distinctive metabolic phenotype characterized by increased fat mass relative to lean mass, altered nutrient partitioning, and greater weight gain per unit of energy intake, in the absence of overt hyperphagia [139]. Notably, MC3R-deficient mice do not exhibit increased food intake even when exposed to high-fat diets, which induce hyperphagia and obesity in MC4R-null models [140].

*Therapeutic Strategies.* Functional defects affecting G-protein signaling and receptor trafficking may represent potential targets for pharmacological intervention, although no MC3R-directed therapies have been developed to date [141]. Modulation of MC3R activity through agonists, ligands, or downstream pathway regulators could potentially influence feeding behavior, energy partitioning, adipose storage, and overall metabolic health. Supporting this concept, animal studies have shown that genetic deletion or pharmacological inhibition of MC3R enhances responsiveness to anorexigenic hormones, suggesting that MC3R modulation may potentiate the effects of existing anti-obesity agents [142].

## 9. Steroid Receptor Coactivator-1 Gene (SRC1)

*Genetic and molecular features.* The *SRC-1* gene, also known as Nuclear Receptor Coactivator-1 (*NCOA1*) (OMIM #602691), is located on chromosome 2q23.3 and encodes the SRC1/NCOA1 protein, a nuclear transcriptional coactivator that binds to nuclear hormone receptors, recruits histone acetyltransferase complexes, promotes histone acetylation, and enhances the expression of target genes [143]. In the context of obesity onset, SRC1 interacts with phosphorylated STAT3 (signal transducer and activator of transcription 3), forming part of a complex that potentiates transcription of POMC [144]. Endocrine features may be influenced by functional SRC1 variants affecting the TSH receptor-α, TSH receptor-β, and estrogen receptor-α [143].

Twenty-three rare heterozygous variants in *SRC1* were identified in a cohort of patients with severe early-onset obesity, with the c.1130C>A (T377N) variant being the most frequent in the non-Finnish European population [145]. Family segregation analyses indicated that rare *SRC1* variants did not follow Mendelian inheritance for severe obesity, suggesting incomplete penetrance. These variants are therefore likely to act as susceptibility modifiers, interacting with additional genetic or environmental factors rather than serving as primary monogenic drivers [146].

*Clinical presentation.* Early-onset obesity and hyperphagia in patients with heterozygous *SRC1* variants are not associated with specific dysmorphic features, and pubertal timing and progression are generally preserved or show only mild advancement [145].

Considering the role of *SRC1* variants in modulating TSH receptor signaling, the observation of partial thyroid hormone resistance and suboptimal TSH suppression in women with autoimmune hypothyroidism, despite adequate levothyroxine therapy, may reflect impaired SRC1-dependent thyroid hormone action. This concept is supported by findings in SRC1-null mice, which exhibit elevated TSH, T4, and T3 levels and require supraphysiologic T3 doses to achieve TSH suppression [145,147]. Endocrine and gynecological-reproductive abnormalities have been reported in cases of SRC1 deficiency, including menstrual dysfunction, polycystic ovary syndrome, endometrial hyperplasia, primary amenorrhea, and hypogonadotropic hypogonadism [145,148]. In addition, SRC1 contributes significantly to bone accrual and mineral homeostasis, primarily through its coactivating function on the estrogen receptor [149]. *SRC1*-related phenotypes include reduced trabecular bone mass and a consequent increased risk of fractures following minimal trauma [150].

Adults with SRC1 variants exhibit markedly increased adipose tissue fibrosis and a potential predisposition to liver fibrosis, without steatosis. Collectively, these findings suggest that SRC1 deficiency promotes accelerated tissue remodeling, highlighting the need for early surveillance for metabolic and hepatic complications in affected individuals [145]. Genetic variants of SRC1 in patients with obesity and hormonal disruption have also been associated with intellectual disability or autism spectrum disorders [151].

*Therapeutic strategies.* Currently, no targeted pharmacological therapies exist for SRC1 deficiency. Setmelanotide, an MC4R agonist, has been administered in selected cases; however, the evidence remains preliminary. Most reported individuals are enrolled in ongoing trials, and robust, long-term clinical data specific to SRC1 variant carriers are not yet available [152]. Additionally, a retrospective analysis of patients harboring heterozygous variants in several obesity-associated genes, including SRC1, who underwent Roux-en-Y gastric bypass (RYGB), suggests that variant carriers may be more prone to postoperative weight regain compared with non-carriers [24].

## 10. Kinase Suppressor of Ras 2 Gene (KSR2)

*Genetic and molecular features.* The Kinase Suppressor of Ras 2 (*KSR2*) gene (OMIM #610737), located on human chromosome 12q24.22–q24.23, encodes a scaffolding protein involved in cellular and mitogenic signaling. *KSR2* integrates mitogenic signals (MAPK pathway) with energetic signals (AMPK pathway), and dysfunction of this gene is associated with pathological metabolic phenotypes, such as early-onset obesity and IR [153,154]. The *KSR2* protein has low intrinsic kinase activity, weakly phosphorylates MEK (MAP2K1) on Ser/Thr residues in vitro, and functions as a scaffold between Raf and MEK, promoting activation of the Ras–Raf–MEK–ERK (MAPK) pathway [153]. Beyond its canonical role in mitogenic signaling, *KSR2* physically associates with the AMP-activated protein kinase (AMPK) complex, a central regulator of cellular energy homeostasis. This interaction enhances AMPK activation and thereby promotes catabolic pathways, including fatty acid oxidation, glucose uptake, and overall energy production [154]. Pathogenic or likely pathogenic *KSR2* variants causing monogenic obesity are very rare in most studied populations [155]. Some variants occur in specific populations; for example, in a Qatari cohort, the intronic variant c.1765-8G>A is significantly more common than in gnomAD, suggesting a possible founder effect [156]. Certain rare variants (rs56214831/p.Arg525Gln) may have a modest population frequency (0.82%) and contribute to weight variation even in the general population, although their effect is moderate and dependent on additional genetic and environmental factors [157]. In monogenic cases, the presence of a *KSR2* variant does not always fully explain the phenotype; familial segregation is not always complete, implying that the variant may increase susceptibility rather than determine the phenotype independently [155].

*Clinical presentation.* Individuals with *KSR2* mutations typically exhibit early-onset hyperphagia, bradycardia, reduced basal metabolic rate, and marked IR, often accompanied by acanthosis nigricans. Notably, other metabolic parameters, including blood pressure, plasma lipids, and adiponectin levels, are generally comparable to those observed in individuals with obesity of other etiologies [155]. Additional features may include IR or a family history of diabetes, hepatic steatosis, vitamin D deficiency, thyroid function abnormalities, and, in some cases, learning difficulties or developmental delay [156].

*Therapeutic strategies.* Several in vitro studies have shown that some effects of *KSR2* mutations, notably impaired oxidation processes, can be partially corrected by metformin, suggesting therapeutic potential. Metformin activates AMPK, and because many of the effects of *KSR2* mutations involve cellular metabolic dysfunction, including glucose and fatty acid oxidation, AMPK activation by metformin appears to partially compensate for these defects [155]. Bariatric surgery has also been used as a therapeutic approach; for example, a 21-year-old patient with severe obesity and compound heterozygous *KSR2* missense mutations (p.Arg224Trp and p.Asp294Glu) showed a significant improvement in BMI, from 50.8 to 34.8 kg/m^2^ (ESPE Abstracts 2019; 92: P1-51). Given that obesity caused by variants such as *KSR2* is at least partly monogenic, genetic correction represents a potential “definitive” cure. Some authors have proposed the future use of CRISPR/Cas9 to repair pathogenic *KSR2* variants or the application of iPSC-derived stem cells to replace defective cells [24].

Table 2 summarizes the genetic and clinical features of autosomal dominant monogenic non-syndromic obesity.

## 11. Conclusions

Autosomal dominant forms of monogenic non-syndromic obesity constitute a clinically heterogeneous subset of early-onset obesity, driven by pathogenic variants that disrupt key nodes of the leptin–melanocortin axis. Despite a growing understanding of their genetic and molecular bases, diagnostic interpretation remains challenging, particularly in the presence of variants of uncertain significance and in cases of incomplete penetrance. Currently, the absence of approved targeted pharmacotherapies leaves affected individuals without precision-based treatment options. Moving forward, the development of mechanism-directed therapeutics for autosomal dominant monogenic obesity will be crucial to improve clinical outcomes, translate advances in gene discovery into personalized interventions, and mitigate hyperphagia and, consequently, the onset of obesity.

## Figures and Tables

**Table 1:** The clinical characteristics may differ according to the specific polymorphisms or variants of the *MC4R* gene.

SNP	Type	Frequency	Clinical/Metabolic Association	Key Notes	Cit.
rs17782313 (allele C)	Common SNP (~188 kb downstream of the main transcript)	~25–40% (depending on ethnicity)	Associated with increased appetite; altered eating behaviors, obesity, hyperglycemia and type 2 diabetes; weak association with cardiovascular disease	Individual effect is modest but relevant at population scale; interacts with environmental factors	[37,38,39,40,41,42]
rs12970134 (allele A)	Common SNP (~153.5 kb downstream of the main transcript)	~15–30% (depending on ethnicity)	Correlated with higher BMI, fat mass, waist circumference, and elevated fasting glucose, triglycerides, and total cholesterol in some studies	Effect appears modifiable by diet (e.g., high-protein/saturated fat) and lifestyle factors	[43,44]
rs13447331 (p.Ser127Leu; c.380C>T)	Rare coding variant missense	Variable frequency	Early-onset severe obesity (loss-of-function)	Severe phenotype; functional impairment of receptor signaling	[45,46]
p.V103Afs*5 (c.308delT: premature stop)	Rare coding variant frameshift	Rare	Very early-onset severe obesity, hyperphagia	Loss-of-function; absent in large public variant databases	[47]
p.Ser19Alafs*34 (c.55del)	Rare coding variant-frameshift	Rare	Very early-onset severe obesity	Effect appears modifiable by environmental and lifestyle factors	[48]
rs13447329 (p.Thr112Met; c.335C>T)	Rare coding variant missense	~0.09% of population	Early-onset severe obesity, hyperphagia, increased height	Suggestive of biased signaling defect (ERK1/2 pathway)	[47]
rs2229616 (p.Val103Ile; c.307G>A)	Missense variant	~2–4%	Variant associated with reduced risk of obesity (protective)	Gain-of-function effect hypothesized; effect modulated by environment/lifestyle	[50,51]
rs52820871 (p.Ile251Leu; c.751A>C)	Missense variant	~2–4%	Strong negative association with childhood obesity and improved outcomes after surgery	Gain-of-function variant; protective effect, but environment/lifestyle still influential	[52]

**Table 2:** Summary of the genetic and clinical features of autosomal dominant monogenic non-syndromic obesity.

Gene (OMIM)	Locus	Obesity Hyperphagia	Main Endocrine/Clinical Characteristics	Behavioral/Neurological/Psychiatrist Characteristics
*MC4R* (#155541)	18q21.32	Yes	Increased linear growth, hyperinsulinemia	Mild learning, mild delay of language, mild intellectual disability, attention-deficit/hyperactivity disorder
*SH2B1* (#608937)	16p11.2	Yes	Insulin resistance, dyslipidemia, high levels of leptin	Autism spectrum disorder, attention-deficit/hyperactivity disorder and mild delay of language.
*SIM1* (#603128)	6q.16.3	Yes	Hypotonia, hypothyroidism, partial diabetes insipidus Prader–Willi-like features	Global developmental delay, reduced concentration, emotional lability, memory deficits.
*GNAS* (#139320)	20q13.32	Yes	Pseudohypoparathyroidism, short stature, round face, subcutaneous calcifications, brachydactyly	Cognitive impairment, development delay, affected motor skills and coordination, seizures
*MRAP2* (#609196)	21q22.11	yes	Hypertension, hyperglycemia and altered adrenal function	Intellectual disability
*MC3R* (#155540)	20q13.2 –q13.3	yes	High leptin levels, altered insulin sensitivity	Intellectual disability
*SRC1* (#602691)	2q23.3	yes	Partial thyroid-hormone resistance, gynecological-reproductive abnormalities, liver fibrosis.	Intellectual disability, autism spectrum disorders
*KSR2* (#610737)	12q24.22–q24.23	yes	Insulin resistance	Learning difficulties

## Data Availability

The original contributions presented in this study are included in the article. Further inquiries can be directed to the corresponding authors.
